# ARPC2 Promotes Pulmonary Fibrosis by Regulating MRTFA Activity Independent of the Canonical ARP2/3 Complex

**DOI:** 10.3390/ijms27062729

**Published:** 2026-03-17

**Authors:** Eun Jo Du, Hyunseong Kim, Seo-Gyeong Bae, Sihyeon An, Kanghyun Ryoo

**Affiliations:** 1 SapiensBio Inc., Seongnam-si 13207, Gyeonggi-do, Republic of Korea; ej.du@sapiensbio.com (E.J.D.); hs.kim@sapiensbio.com (H.K.); sg.bae@sapiensbio.com (S.-G.B.); 2 YD Global Life Science Co., Ltd., Seongnam-si 13207, Gyeonggi-do, Republic of Korea

**Keywords:** idiopathic pulmonary fibrosis, ARPC2, fibroblast-to-myofibroblast transition, MRTFA

## Abstract

Idiopathic pulmonary fibrosis (IPF) is a chronic, progressive lung disease characterized by the pathological accumulation of collagen-rich extracellular matrix, resulting in irreversible lung remodeling and respiratory failure. The incomplete understanding of IPF pathogenesis has hindered the development of effective therapeutics. Here, we investigate the mechanism by which the actin-related protein 2/3 complex subunit 2 (ARPC2) contributes to the fibrotic response in lung fibroblasts. Modulating of ARPC2 expression levels altered the expression of profibrotic genes, including α-smooth muscle actin (*ACTA2*), in TGF-β1-treated MRC-5 cells at the transcriptional level. We further show that ARPC2 regulates the TGF-β1-mediated nuclear translocation of myocardin-related transcription factor-A (MRTFA), a central driver of fibrotic gene induction. Our data indicate that ARPC2 plays a distinct role in profibrotic gene expression and MRTFA nuclear localization, distinguishing its function from other components of the actin-related protein 2/3 (ARP2/3) complex. Furthermore, ARPC2 appears to modulate the TGF-β1-dependent formation of MRTFA/G-actin complexes. Finally, transcriptomic analysis of cells depleted of ARPC2, ACTR2, or MRTFA revealed that ARPC2 and MRTFA co-regulate a specific repertoire of fibrotic genes. These observations support a profibrotic function for ARPC2 during fibroblast-to-myofibroblast transition (FMT), highlighting it as a potential therapeutic target for IPF.

## 1. Introduction

Idiopathic pulmonary fibrosis (IPF) is a chronic, progressive interstitial lung disease characterized by excessive fibrotic remodeling of the pulmonary architecture, ultimately resulting in severe ventilatory impairment and respiratory failure. The clinical course of IPF remains poor, with 5-year survival rates of approximately 30%, comparable to those of several malignancies [1]. Although a few antifibrotic medications—pirfenidone, nintedanib and nerandomilast—have demonstrated efficacy in slowing functional decline, these agents have not substantially improved overall survival or reversed fibrotic pathology [2,3,4,5]. At present, lung transplantation remains the only definitive treatment option, though its availability is severely limited by donor shortage and surgical risks. These limitations underscore the need for the development of more effective treatments for IPF.

The precise molecular mechanisms underlying IPF pathogenesis remain incompletely understood. However, the differentiation and sustained activation of myofibroblasts have been identified as central drivers of disease progression [6,7,8]. Following exposure to injurious stimuli, including repetitive epithelial damage and inflammatory mediators, pro-fibrotic factors—e.g., transforming growth factor-β (TGF-β), platelet-derived growth factor (PDGF), and connective tissue growth factor (CTGF)—orchestrate myofibroblast differentiation [9,10,11]. This differentiation occurs predominantly through two pathways: epithelial-mesenchymal transition (EMT) and fibroblast-to-myofibroblast transition (FMT). The TGF-β signaling cascade serves as the primary driver of these pathways, promoting α-smooth muscle actin (*ACTA2*) expression and extracellular matrix synthesis [12]. While EMT has been extensively studied as a therapeutic target, the molecular mechanisms governing FMT remain less well characterized, representing an important area for further investigation.

The actin-related protein 2/3 (ARP2/3) is a seven-subunit regulator of actin polymerization and branching in eukaryotic cells [13,14]. The mechanistic basis for ARP2/3-mediated fibrogenesis is linked to the close relationship between actin cytoskeleton dynamics and profibrotic transcriptional programs. Myofibroblast differentiation requires the formation of stress fibers, which are assembled through coordinated actin polymerization [15,16]. Actin branching networks also regulate the subcellular localization and transcriptional activity of profibrotic factors including myocardin-related transcription factor-A (MRTFA) and Yes-associated protein (YAP)/transcriptional coactivator with PDZ-binding motif (TAZ) [17,18]. Specifically, actin polymerization facilitates MRTFA-dependent transcription: depletion of the cytoplasmic pool of monomeric globular actin (G-actin) leads to dissociation of the MRTFA/G-actin complex, enabling MRTFA nuclear translocation. In the nucleus, MRTFA cooperates with serum response factor (SRF) to drive transcription of profibrotic genes including *ACTA2*, *COL1A1*, and *CTGF* [15,16,17,18].

Despite the extensive characterization of the ARP2/3 complex, the distinct functional contributions of individual ARP2/3 subunits in pathophysiological contexts remain largely unexplored. However, actin-related protein 2/3 complex subunit 2 (ARPC2) was previously reported to regulate collagen expression in lung fibroblasts and is negatively regulated by Forkhead Box F1 (FOXF1), a transcription factor reportedly reduced in IPF [19]. In addition, increased expression of ARPC2 was reported in the GDS4279 IPF patients lung tissue profile [20]. Here, we investigated the individual role of ARPC2, distinguishing its function from that of actin-related protein 2 (ACTR2), an ATP-binding component of the ARP2/3 complex where actin nucleation initiates. We evaluated the effects of ARPC2 on fibrogenesis and investigated the associated molecular pathway, focusing on its role in regulating MRTFA nuclear translocation through the modulation of G-actin association during TGF-β1 stimulation. Together, our findings implicate ARPC2 as a potential therapeutic target for IPF.

## 2. Results

### 2.1. ARPC2 Knockdown Attenuates the Fibrotic Response in TGF-β1-Stimulated MRC-5 Cells

**Figure 1:**
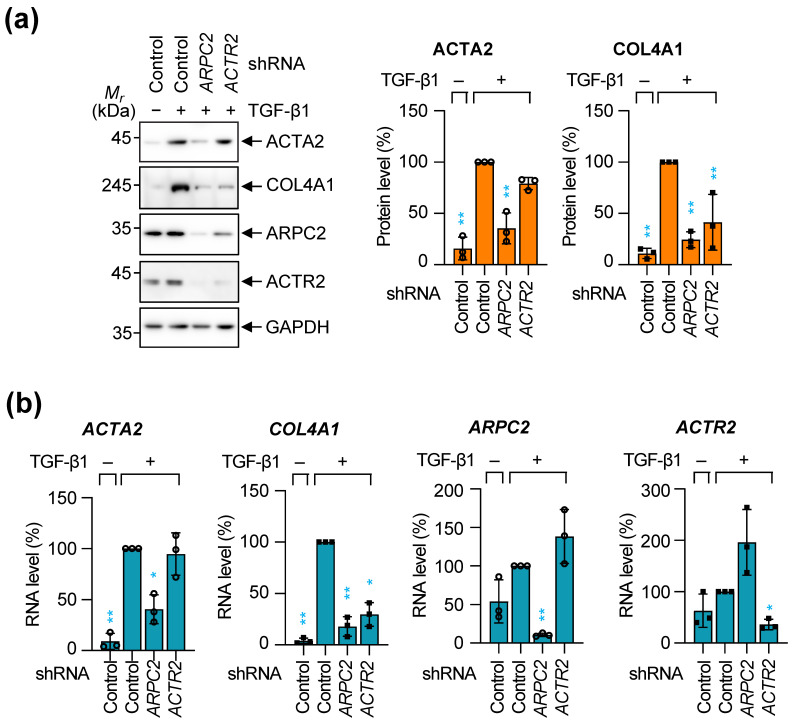
ARPC2 knockdown inhibits TGF-β1 induced fibrotic response in MRC-5 cell. MRC-5 cells were infected with lentiviruses expressing shRNA targeting ARPC2 or ACTR2 mRNA, followed by 72-h incubation under serum-starved conditions with TGF-β1 to evaluate fibrotic attenuation. (**a**) Western blot analysis using primary antibodies against of ACTA2, COL4A1, ARPC2, ACTR2, and GAPDH. Representative bands are shown in the left panel. Quantification of fibrosis markers (right panel) is normalized to the GAPDH and compared to TGF-β1-treated group (100%). Mean ± SD (*n* = 3; ** *p* < 0.01). (**b**) RNA levels of *ACTA2*, *COL4A1*, *ARPC2* and *ACTR2* were normalized to the *GAPDH* and compared to TGF-β1-treated group (100%). Mean ± SD (*n* = 3; * *p* < 0.05; ** *p* < 0.01). Statistical significance was determined by one-way ANOVA followed by Dunnett’s multiple comparisons test.

To investigate the role of individual ARP2/3 subunits in modulating human lung fibrosis, we achieved small hairpin RNA (shRNA)-mediated knockdown of *ARPC2* and *ACTR2* in MRC-5 cells using lentiviral transduction. To distinguish the novel, non-canonical function of ARPC2 from canonical actin nucleation, we utilized *ACTR2* knockdown as a functional control for the intact ARP2/3 complex. ACTR2 encodes an ATP-binding subunit that, together with ACTR3, forms the catalytic core required for de novo actin filament nucleation and the generation of branched actin networks [21,22]. Transduced cells were subsequently incubated in the presence or absence of TGF-β1 to induce the fibrotic response. As expected, TGF-β1 treatment significantly upregulated ACTA2 and COL4A1 at the protein and RNA levels in the cells transduced with negative control shRNA (Figure 1). TGF-β1 treatment had a minimal effect on the expression of ARPC2 and ACTR2. We also observed that the depletion of either ARPC2 or ACTR2 led to a reduction in the protein level of each other subunit but not the transcript (Figure 1a), consistent with the well-documented interdependence of these proteins [23,24].

Induction of *ACTA2* and *COL4A1* by TGF-β1 in *ARPC2*- or *ACTR2*-depleted cells was significantly inhibited, however, the RNA level of *ACTA2* was repressed only in the *ARPC2*-depleted cells (Figure 1a,b). Given that ACTR2 is a pivotal component of the ARP2/3 complex [25,26], and that *ACTR2*-depleted cells have reduced protein levels of other ARP2/3 components, we speculated that ARPC2 regulates the TGF-β1-mediated induction of *ACTA2* at the transcriptional step via a mechanism distinct from the ARP2/3 complex.

We further investigated the effect of *ARPC2*- or *ACTR2*-knockdown on stress fiber formation. Immunocytochemistry using an antibody specific to endogenous ACTA2 showed that the depletion of either subunit significantly reduced the fluorescence intensity of ACTA2 in TGF-β1-treated MRC-5 cells (Supplementary Figure S1a). The discrepancy observed between the immunocytochemistry and immunoblotting results suggests that ARPC2 depletion represses both the expression and structural assembly of ACTA2, whereas ACTR2 depletion is limited to impairing the structural assembly of ACTA2 into stress fibers—consistent with the canonical role of the ARP2/3 complex in actin polymerization.

### 2.2. ARPC2 Overexpression Induces Fibrotic Response in MRC-5 Cells

**Figure 2:**
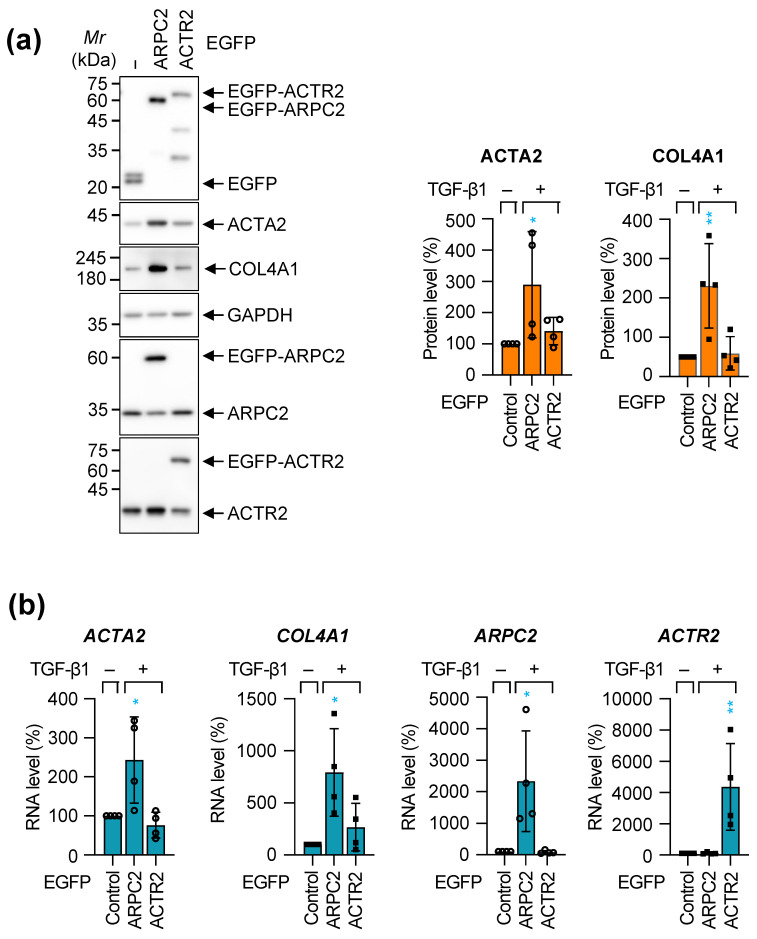
Ectopic overexpression of ARPC2 induces fibrotic response in MRC-5 cells. MRC-5 cells were infected with adenoviruses expressing EGFP-fused ARPC2 or ACTR2 and incubated for 72 h under serum-starved conditions to assess fibrotic induction. (**a**) Western blot analysis using primary antibodies against of EGFP, ACTA2, COL4A1, GAPDH, ARPC2, and ACTR2. Representative bands are shown in the left panel. Quantification of fibrosis markers (right panel) is normalized to GAPDH and compared to the control group (100%). Mean ± SD (*n* = 4; * *p* < 0.05; ** *p* < 0.01). (**b**) RNA levels of *ACTA2*, *COL4A1*, *ARPC2* and *ACTR2* were normalized to the *GAPDH* and compared to the control group (100%). Mean ± SD (*n* = 4; * *p* < 0.05; ** *p* < 0.01). Statistical significance was determined by one-way ANOVA followed by Dunnett’s multiple comparisons test.

To distinguish the individual function of ARPC2 in the fibrotic response following our knockdown observations, we examined the effects of overexpressing either ARPC2 or ACTR2 in fibrotic response of MRC-5 cells without TGF-β1 stimulation. This was achieved using adenovirus vectors encoding *EGFP*-fused *ARPC2* or *ACTR2* coding region, which were then transduced into the MRC-5 cells. Unlike the reciprocal protein reduction observed in the knockdown experiment (Figure 1a), neither ARPC2 nor ACTR2 overexpression led to mutual induction of the other subunit’s expression (Figure 2a,b).

Overexpression of ARPC2 markedly increased both protein and transcript levels of *ACTA2* and *COL4A1* in MRC-5 cells, in stark contrast, ACTR2 overexpression resulted in levels comparable to the control (Figure 2a,b). This finding suggests that the fibrotic response driven by ARPC2 overexpression is attributable to ARPC2 itself, operating distinctly from the ARP2/3 complex. Furthermore, immunocytochemistry showed that ARPC2 overexpression significantly increased the ACTA2 fluorescent intensity, while the effect of ACTR2 overexpression remained minimal, although statistically significant (Supplementary Figure S1b).

Collectively, these data support a role for ARPC2 as a fibrosis-inducing factor, operating through a distinct pathway largely independent of its role within the canonical ARP2/3 complex, to modulate pro-fibrotic factor expression and stress fiber formation.

### 2.3. ARPC2 Promotes the Fibrotic Response by MRTFA Nuclear Translocation

To further explore the underlying mechanism of ARPC2 in the fibrotic response, we investigated potential interactions with pro-fibrotic pathways, particularly those involving myocardin-related transcription factor A (MRTFA). MRTFA is a key regulator of myofibroblast activation in various fibrotic diseases, including cardiac and pulmonary fibrosis, and its transcriptional activity is intimately linked to the expression of pro-fibrotic genes [27,28,29].

**Figure 3:**
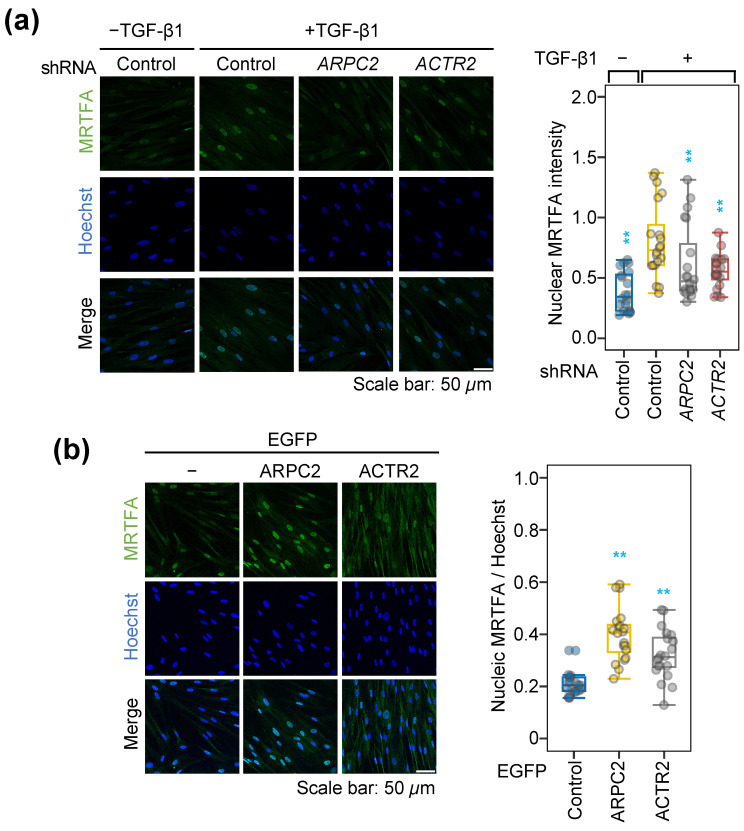
ARPC2 modulates MRTFA nucleocytoplasmic shuttling. Experiments involving ARPC2 and ACTR2 knockdown and overexpression in MRC-5 cells under serum-starved conditions elucidated their roles in MRTFA nucleocytoplasmic shuttling. (**a**) Knockdown of ARPC2 or ACTR2 followed by 72-h incubation with TGF-β1. Nuclear localization of MRTFA was detected with immunocytochemistry and quantified as nuclear MRTFA (green) intensity normalized to Hoechst (blue) intensity. Box plots show median, IQR, and 1.5× IQR whiskers; individual data points are jittered (*n* = 4; ** *P*_adj_ < 0.01; compared to TGF-β1 treated control). (**b**) Overexpression of EGFP-fused ARPC2 or ACTR2 (72 h). Nuclear localization of MRTFA was quantified as in (**a**) with minor adjustment, by using TRITC-conjugated antibody to detect MRTFA, which shown in pseudo color green. Box plots show median, IQR, and 1.5× IQR whiskers; individual data points are jittered (*n* = 4; ** *P*_adj_ < 0.01; compared to control). Quantified image data were analyzed using a generalized linear mixed model (GLMM) with FDR correction for multiple comparisons to the control group (see Materials and Methods for details).

Given the importance of MRTFA in fibrosis, we hypothesized that ARPC2 regulates MRTFA nuclear localization or activity to drive fibrosis. To verify this hypothesis, we examined MRTFA localization in MRC-5 cells stimulated with TGF-β1. Upon TGF-β1 treatment, MRTFA exhibited a clear nuclear accumulation, confirming that pro-fibrotic gene induction in MRC-5 cells occurs via transcriptional activation through MRTFA (Figure 3a). Consistent with our previous observation for pro-fibrotic gene expression, depletion of *ARPC2* or *ACTR2* impaired MRTFA nuclear translocation (Figure 1 and Figure 3a). Furthermore, ARPC2 overexpression significantly induced MRTFA nuclear translocation even without TGF-β1 stimulation, while the effect of ACTR2 overexpression effect remained minimal, although statistically significant (Figure 3b). Collectively, these findings suggest that ARPC2 functions as a key factor to induce pro-fibrotic gene expression by directly regulating MRTFA nuclear translocation.

To uncover the underlying molecular mechanism of ARPC2-driven MRTFA nuclear localization, we explored the interaction dynamics between MRTFA and G-actin, which is a well-documented molecular mechanism of regulating MRTFA nucleocytoplasmic shuttling [30,31]. In normal conditions, MRTFA associates with five G-actin molecules through the three RPEL motifs in the N-terminal region of MRTFA, shielding the nuclear localization signal (NLS) necessary for nuclear transport (Supplementary Figure S2a) [31,32,33]. In response to fibrotic stimuli such as TGF-β1, MRTFA dissociates from G-actin, thereby facilitating its nuclear translocation by exposing its NLS region and inducing pro-fibrotic gene expression.

**Figure 4:**
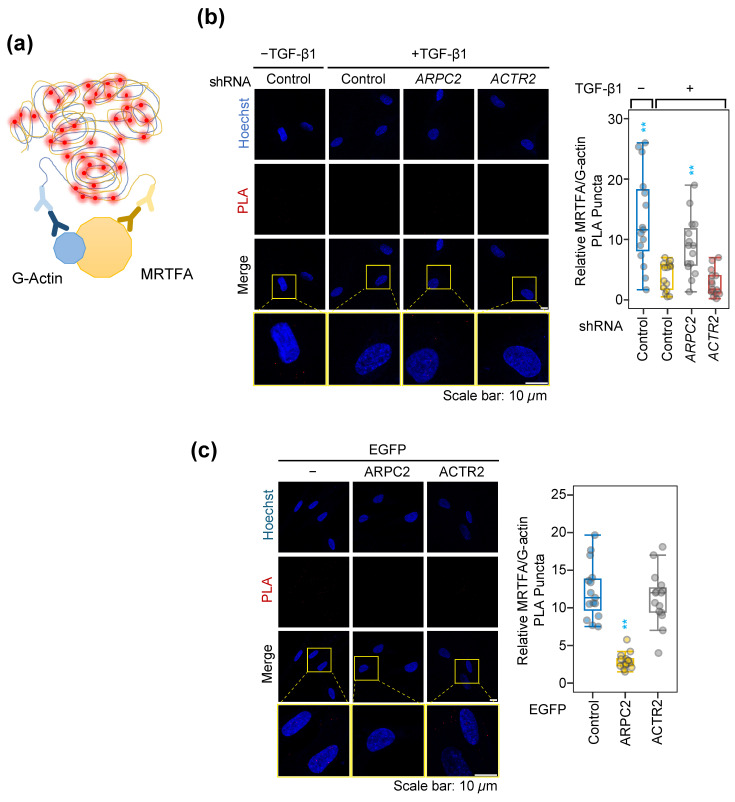
ARPC2 disrupts MRTFA/G-actin interaction to facilitate nuclear translocation. (**a**) Schematic diagram illustrating the principle of in-situ PLA used to detect MRTFA/G-actin interaction. Schematic diagram was generated using Microsoft Powerpoint. (**b**) Knockdown of ARPC2 or ACTR2 followed by 72-h incubation with TGF-β1. MRTFA/G-actin interactions were assessed via PLA, with puncta (red) counts normalized to the number of nuclei (Hoechst; blue). Box plots show median, IQR, and 1.5× IQR whiskers; individual data points are jittered (*n* = 3; ** *P*_adj_ < 0.01; compared to TGF-β1 treated control). (**c**) Overexpression of EGFP-fused ARPC2 or ACTR2 (72 h under serum starvation). MRTFA/G-actin interactions were assessed via PLA, with puncta (red) counts normalized to the number of nuclei (Hoechst; blue). Box plots show median, IQR, and 1.5× IQR whiskers; individual data points are jittered (*n* = 3; ** *P*_adj_ < 0.01; compared to control). Quantified image data were analyzed using a generalized linear mixed model (GLMM) with FDR correction for multiple comparisons to the control group (see Materials and Methods for details).

Using an in-situ proximity ligation assay (PLA), we observed a significant reduction in the MRTFA/G-actin interaction under TGF-β1 stimulation conditions in MRC-5 cells (Figure 4a,b). This reduction is clearly consistent with the established regulatory mechanism of MRTFA in the fibrotic response, translocating to the nucleus by disengaging from G-actin. Notably, *ARPC2* depletion abolished the dissociation of MRTFA/G-actin interaction in TGF-β1 stimulated MRC-5 cells, maintaining interaction at a similar level to unstimulated control (Figure 4b). In addition, ARPC2 overexpression was sufficient to dissociate MRTFA/G-actin interaction without TGF-β1 stimulation (Figure 4c), consistent with previous observations in pro-fibrotic marker expression and MRTFA nuclear localization (Figure 2 and Figure 3b). In contrast, neither ACTR2 knockdown nor overexpression affected MRTFA/G-actin interaction (Figure 4b,c). Collectively considering the marginal effects of *ACTR2* modulation on pro-fibrotic marker gene expression and MRTFA nuclear translocation, ACTR2 does not appear to directly regulate MRTFA activity during the fibrotic response (Figure 1, Figure 2, Figure 3 and Figure 4). These findings further highlight the distinct molecular mechanism of ARPC2 in fibrosis compared to other ARP2/3 components.

To gain a deeper understanding of the underlying mechanism regulating MRTFA/G-actin interaction by ARPC2, we investigated whether ARPC2 physically interacts with MRTFA in response to TGF-β1 stimulation. Using in-situ PLA, we measured interaction dynamics between MRTFA and ARPC2, ACTR2 or ARPC4-a neighbor subunit located right next to ARPC2 in ARP2/3 complex. Interestingly, the PLA signal between MRTFA and ARPC2 was significantly detected in TGF-β1 stimulated MRC-5 cells, while interactions with ARPC4 or ACTR2 remained at basal levels (Supplementary Figure S2b). Moreover, in vitro binding assay using recombinant ARPC2 and MRTFA N-terminus showed direct binding in a cell-free condition (Supplementary Figure S2c). This result provides evidence for a novel molecular mechanism of ARPC2 in MRTFA activation to induce pro-fibrotic gene expression in the presence of fibrosis stimuli, which appears independent of the other components of ARP2/3 complex.

### 2.4. Transcriptomic Analysis Reveals ARPC2-Dependent Regulation of MRTFA Response Genes

**Figure 5:**
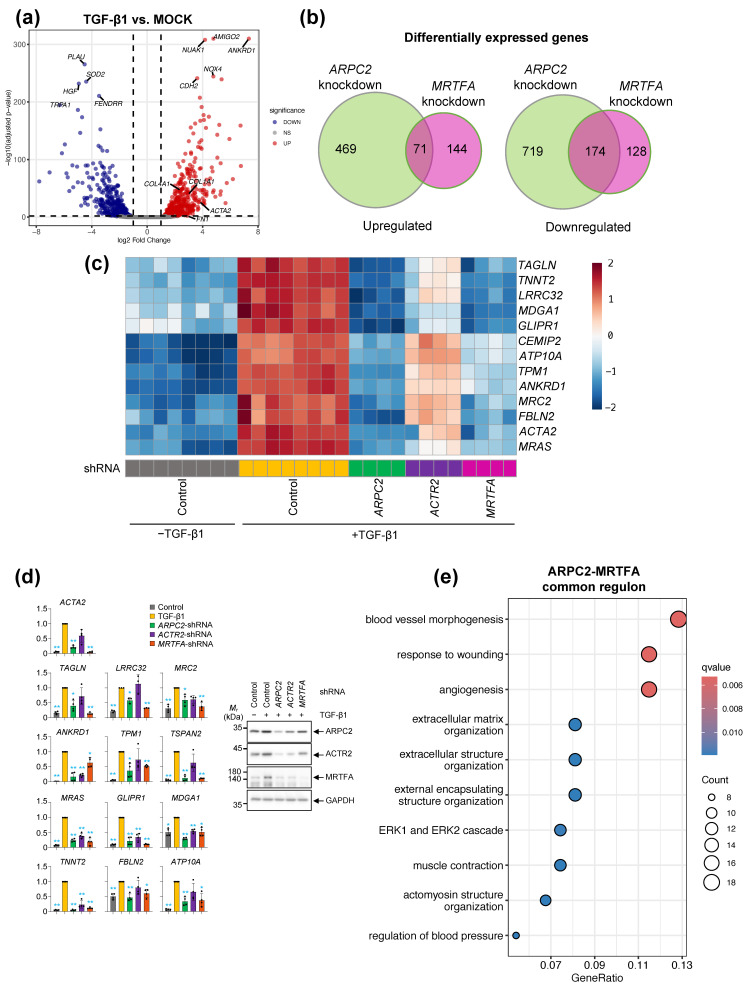
Transcriptomic analysis reveals a shared fibrotic regulon between ARPC2 and MRTFA. Transcriptomic analysis was performed in the MRC-5 cells by comparing (i) pre- and post- TGF-β1 treatment groups (control; *n* = 8 per group) and (ii) lentiviral transduction and TGF-β1 treatment condition (*n* = 4 per group). All DEGs were filtered using cut-off values of absolute log_2_ fold change > 1 and FDR < 0.01. (**a**) Volcano plot displaying differential gene expression between TGF-β1-treated and unstimulated control cells. Significant DEGs are highlighted in red (upregulated) and blue (downregulated). The top 5 most significantly altered genes and pro-fibrotic genes (*ARPC2*, *FN1*, *COL1A1* and *COL4A1*) are labeled. (**b**) Venn diagram illustrating the overlap of DEGs between ARPC2- and MRTFA-knockdown groups, categorized by their response to TGF-β1 (upregulated or downregulated). Gene counts for each category are indicated. (**c**) Heatmap depicting the relative expression levels of key fibrotic response genes across all experimental groups. Pre- and post-TGF-β1 treatment groups were indicated by dash line. (**d**) Validation of gene expression profiles from (**c**) using RT-qPCR. Dots represent individual biological replicates. Mean ± SD (*n* = 4; * *p* < 0.05; ** *p* < 0.01; compared to TGF-β1 treated control). Representative bands of Western blot analysis using primary antibodies against of ARPC2, ACTR2, MRTFA, and GAPDH are shown in the right panel. (**e**) Gene Ontology (GO) enrichment analysis of biological processes associated with the genes commonly downregulated by both ARPC2 and MRTFA depletion.

To advance our understanding of the mechanistic role of ARPC2 enhancing MRTFA nuclear translocation, we performed genome-wide transcriptomic analysis of *ARPC2*-, *ACTR2*-, and *MRTFA*-depleted MRC-5 cells. As expected in our previous observation, the transcriptional profile from RNA-seq showed significant induction of pro-fibrotic marker and fibrosis related genes by TGF-β1 stimulation, such as *ACTA2* and collagen family (Figure 5a and Supplementary Table S1). Consistent with our previous observations, *ARPC2* depletion significantly repressed the expression of TGF-β1-induced fibrosis related genes, while *ACTR2* depletion showed a lesser or non-significant effect (Figure 5c). As expected, *MRTFA* depletion significantly reduced those gene expressions, with a similar level of unstimulated control (Figure 5c). In the qPCR experiment we confirmed the expression level of TGF-β1-induced fibrosis related genes, and revealed that *ACTA2*, *TAGLN*, *LRRC32*, *MRC2*, *TPM1*, *TSPAN2*, *FBLN2*, and *ATP10A* were repressed by *ARPC2*- and *MRTFA*-depletion, respectively while *ACTR2*-depletion was not (Figure 5d).

Comparing differentially expressed genes (DEG) revealed ARPC2- and MRTFA-knockdown dependent DEGs shown partially overlapped, with common 71 upregulated and 174 downregulated genes (Figure 5b). As our previous investigation revealed the molecular action of ARPC2 which regulating MRTFA nuclear translocation, we analyzed the ARPC2-MRTFA common downregulated DEGs and revealed were significantly enriched in Gene Ontology (GO) of fibrosis-related categories, including wound healing and extracellular matrix organization (Figure 5e, Figure S3 and Supplementary Table S1). Notably, DEGs identified between the ARPC2 and ACTR2 knockdown groups showed significant enrichment in fibrosis-related biological processes, suggesting a distinct regulatory role for ARPC2 (Supplementary Figure S3d). This finding suggests a systematic regulation, within ARPC2-dependent transcriptomic regulation is deeply reliant on MRTFA-dependent pathway, which regulates pro-fibrotic gene expression and myofibroblast transition.

**Figure 6:**
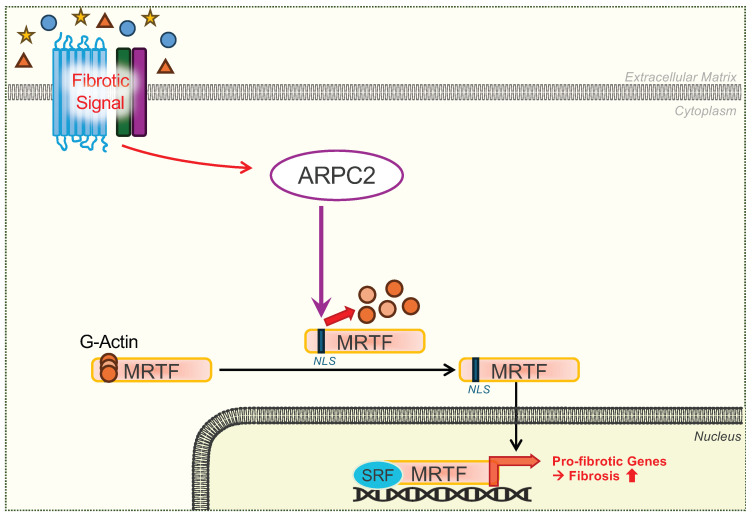
Proposed schematic mechanism of ARPC2-dependent MRTFA regulation in pro-fibrotic gene expression. Upon TGF-β1 stimulation, ARPC2 facilitates the crucial dissociation of MRTFA from G-actin, thereby promoting its nuclear translocation and enhancing the transcriptional activity of pro-fibrotic genes. Schematic diagram was generated using Microsoft Powerpoint.

These findings indicate that ARPC2 plays a crucial role in regulating fibrosis by modulating MRTFA’s nuclear localization through the disruption of MRTFA/G-actin interaction. By facilitating MRTFA’s nuclear translocation, ARPC2 significantly enhances MRTFA-mediated transcriptional activation of pro-fibrotic genes, thereby driving the fibrotic response (Figure 6). This mechanistic insight provides a profound and clearer understanding of how ARPC2 acts as a central modulator in the complex signaling pathways underlying fibrosis.

In summary, our results unveil a novel and critical mechanism wherein ARPC2 drives fibrosis through a distinct molecular pathway, independent of its canonical role within the ARP2/3 complex. This discovery highlights ARPC2 as a pivotal regulator in the fibrotic process, suggesting its potential as a therapeutic target for fibrosis diseases such as IPF.

## 3. Discussion

Fibrotic diseases, including IPF, represent a significant global health challenge characterized by aberrant wound healing responses. Given their profound biological complexity, a deeper understanding of their underlying pathology is essential for developing effective therapeutic strategies. Myofibroblasts, defined by their prominent induction of ACTA2 stress fibers and the tissue stiffness, are central contributors to chronic fibrosis when excessively activated or abnormally differentiated. This pathological differentiation is largely attributed to processes like epithelial-mesenchymal transition (EMT) or fibroblast-myofibroblast transition (FMT) [12,34]. While most current IPF therapeutics have primarily focused on blocking EMT, targeting FMT has remained less explored due to a persistent lack of clarity regarding its intricate molecular mechanisms. Our study addresses this critical gap by identifying a novel fibrosis-inducing factor, ARPC2, and elucidating its molecular mechanism of action: the induction of pro-fibrotic gene through MRTFA activation in human fibroblast MRC-5 cells.

It is widely accepted that the induction of chronic fibrosis typically necessitates stimulation by pro-fibrotic cytokines such as transforming growth factor-beta (TGF-β), tumor necrosis factor-alpha (TNF-α), platelet-derived growth factor (PDGF), and interleukins [12]. Our findings demonstrate that ARPC2 knockdown significantly reduced fibrotic response induced by TGF-β1 (Figure 1). Furthermore, ARPC2 overexpression alone was sufficient to induce a significant fibrotic response without any stimuli, suggesting that ARPC2 contributes for the fundamental pathway for driving fibrotic response (Figure 2). While currently approved therapies for IPF predominantly target upstream signaling pathways to block the fibrosis, ARPC2-targeting therapy would give advance to achieve down-stream regulation of fibrosis by regulating the major fibrosis activator, MRTFA (Figure 3, Figure 4 and Figure 5). We revealed that ARPC2 regulates MRTFA functionality by regulating its nuclear shuttling (Figure 3, Figure 4 and Figure 5). This evidence suggests that ARPC2 plays a pivotal role in driving pathological FMT progression.

Previous investigations into the ARP2/3 complex in the context of fibrosis have primarily reported its function in mediating the assembly of actin stress fibers and facilitating myofibroblast differentiation [16,18,35]. In contrast, our findings reveal a previously unrecognized molecular mechanism by which ARPC2 orchestrates fibrotic responses through a pathway that is distinct from the canonical actin nucleation machinery of the ARP2/3 complex. We observed a divergence of ACTA2 protein level between immunoblotting (total protein) and immunocytochemistry (structural intensity) in ACTR2-depleted cells (Figure 1a and Figure S1a). This supports the notion that ACTR2, as an essential catalytic subunit for initiating actin polymerization within the ARP2/3 complex, contributes for actin polymerization and stress fiber formation (Supplementary Figure S1a), but—unlike ARPC2—does not govern the transcriptional regulation of ACTA2 mRNA (Figure 1a). Furthermore, ACTR2 perturbation exerted only marginal effects on pro-fibrotic genes without modulating MRTFA/G-actin dynamics (Figure 2, Figure 5 and Figure S1b), the fibrotic response driven by ARPC2 appears to operate independently of conventional actin stress-fiber remodeling mechanisms. Instead, our data demonstrate that ARPC2 governs MRTFA nuclear translocation by directly facilitating its dissociation from G-actin (Figure 3, Figure 4 and Figure S2). Corroborating this insight into the mechanism, transcriptome profiling revealed that MRTFA-responsive genes underwent significant downregulation following ARPC2 depletion, whereas ACTR2 knockdown failed to elicit comparable transcriptional alterations (Figure 5).

The activation of MRTFA is intimately linked to cytoskeletal dynamics and FMT. In response to pro-fibrotic signals like TGF-β1, MRTFA shuttles into the nucleus, where it activates the transcription of pro-fibrotic genes in conjunction with serum response factor (SRF) [32,36,37,38]. MRTFA contains three N-terminal RPEL motifs that associate with G-actin. This association, tightly coupled with its nuclear localization signal (NLS), effectively blocks nuclear import by preventing importin-α/β-dependent transport (Supplementary Figure S2a) [31]. Our experiments show that ARPC2 modulation regulates the MRTFA/G-actin interaction and MRTFA nuclear import (Figure 3 and Figure 4). This suggests a novel molecular mechanism where ARPC2 directly induces the dissociation of the MRTFA/G-actin interaction. In addition, in-situ PLA revealed that MRTFA physically interacted with ARPC2 upon TGF-β1 stimulation, while showing no interaction with ARPC4 or ACTR2 (Supplementary Figure S2b). Based on these observations, we posit that the TGF-β1 signal enhances the interaction between ARPC2 and MRTFA, leading to the dissociation of G-actin from MRTFA’s RPEL motifs. The lack of PLA signal between MRTFA and ACTR2 or ARPC4 further supports that the ARP2/3 complex itself is unlikely to be involved in this specific MRTFA activation pathway. Collectively, these findings establish that ARPC2 functions as a unique molecular regulator of fibrotic response through a non-canonical, actin nucleation-independent mechanism that selectively modulates MRTFA transcriptional activity, thereby distinguishing its biological function from the structural role of the conventional ARP2/3 complex.

Given that ARPC2 is ubiquitously expressed in most tissues, how is it maintained in an inactive state under normal conditions, yet aberrantly activated during fibrosis? We observed a tight interdependence between ARP2/3 components, where targeted knockdown of ARPC2 or ACTR2 led to a specific reduction in the protein level of the reciprocal subunit while transcript levels remained unaffected (Figure 1). This phenomenon, where the loss of a single subunit leads to the destabilization of the entire ARP2/3 subunits, has been previously documented across various eukaryotic species, including *Saccharomyces cerevisiae* [39], *Arabidopsis thaliana* [40], *Mus musculus* [41,42] and *Homo sapiens* [43]. The exact mechanisms underlying this stoichiometric interdependence are not yet fully understood, but it appears that the absence of core structural subunits severely impairs complex association [40]. Integrating these observations with our findings, we propose that ARPC2 may exist in two distinct functional pools within cells: (i) a stable form associated with the ARP2/3 complex, primarily involved in its canonical function, the actin remodeling; and (ii) a more unstable, free or dissociated form that becomes activated upon fibrotic stimuli to drive FMT. The inherent instability (or rapid turnover) of this fibrosis-driving ARPC2 pool may be crucial for orchestrating a controlled wound healing process, thereby preventing progression to chronic pathological states. Conversely, the aberrant accumulation of ARPC2 leads to an uncontrolled, sustained fibrotic drive.

In conclusion, our study provides a significant advance in understanding FMT, a critical process underlying IPF. We have identified the ARPC2-driven FMT pathway through MRTFA activation as a novel mechanism in this pathology. Given the high evolutionary conservation of ARPC2—with human and murine proteins sharing >99% sequence identity—alongside the conserved regulatory N-terminal region of MRTFA, we anticipate this signaling axis is highly generalizable to mammalian systems. To validate these findings, future in vivo studies using lung fibrosis model will be essential, such as bleomycin-induced pulmonary fibrosis models employing lung fibroblast-specific conditional *Arpc2* knockout mice or ARPC2-targeting therapeutics. Ultimately, these findings not only illuminate ARPC2 as a key regulator of lung fibrosis but also provide crucial insights into the broader regulation of fibrotic responses, thereby opening a new approach for the identification of novel therapeutic targets to treat chronic fibrotic diseases.

## 4. Materials and Methods

### 4.1. Cell Culture and TGF-β1 Stimulation

Human lung fibroblast cells, MRC-5 (ATCC, CCL-171), were utilized at passages under 12. Cells were maintained in Minimum Essential Medium (MEM; Welgene, Gyeongsan, Republic of Korea, LM007-54) supplemented with 10% fetal bovine serum (FBS; Gibco, Waltham, MA, USA, 16000-044) and 1× Antibiotic-Antimycotic (Gibco, Waltham, MA, USA, 15240-062). To induce fibrosis, the culture medium was replaced with serum-reduced MEM containing 0.5% FBS and stimulated with 5 ng/mL recombinant human TGF-β1 (R&D Systems, Minneapolis, MN, USA, 240-B). For unstimulated negative control, cells received serum-reduced MEM only.

### 4.2. Adenovirus and Lentivirus

Adenovirus and lentivirus vectors employed in this study were constructed and packaged by VectorBuilder (Chicago, IL, USA). Chimeric Ad5/F35 adenovirus vectors were constructed of N-terminal EGFP-fused human ARPC2 (VectorBuilder, Chicago, IL, USA; VB231015-1305rdp), ACTR2 (VectorBuilder, Chicago, IL, USA; VB231015-1308whh), or EGFP alone (VectorBuilder, Chicago, IL, USA; VB010000-9301bcw) [44]. Adenoviral infection involved a 6-h incubation in culture medium containing the indicated adenovirus, followed by replacement with serum-reduced MEM.

Lentiviral particles encoding shRNA targeting ARPC2 (VectorBuilder, Chicago, IL, USA; VB240708-1692edp), ACTR2 (VectorBuilder, Chicago, IL, USA; VB240708-1700mbm), MRTFA (VectorBuilder, Chicago, IL, USA; VB241125-1652qag), or a non-targeting scramble control (VectorBuilder, Chicago, IL, USA; VB010000-9541pqu) were obtained. Lentiviral infection was performed by incubating cells with the indicated lentivirus and 8 µg/mL polybrene for 48 h prior to fibrosis stimulation.

### 4.3. Quantitative Real-Time PCR (qRT-PCR)

qRT-PCR was performed to quantify the mRNA levels of selected human genes in MRC-5 cells. Total RNA was extracted using TRIzol (Thermo Fisher Scientific, Waltham, MA, USA, 15596018). cDNA was synthesized from 1 µg of total RNA using a reverse transcriptase (Thermo Fisher Scientific, Waltham, MA, USA, EP0441) in a 20 µL reaction, following the manufacturer’s instructions. cDNA quantification for each gene was performed by qRT-PCR using LightCycler^®^ 480 SYBR Green (Roche, Basel, Switzerland) and primers detailed in the below. Relative gene mRNA levels were calculated using the 2^−ΔΔCt^ method and normalized to GAPDH expression. All qPCR reactions were performed in triplicate, and the average of the three replicates was used for analysis.

qPCR primers used in this study: GAPDH: 5′-TGGCAAATTCCATGGCACC-3′ and 5′-AGAGATGATGACCCTTTTG-3′; ACTA2: 5′-AGACATCAGGGGGTGATGGT-3′ and 5′-CATGGCTGGGACATTGAAAG-3′; COL4A1: 5′-AGGATCTGTTGGTGGAATGGGCT-3′ and 5′-AATGCTTCCTTTTTCTCCCTTCTC-3′; ARPC2: 5′-GAACCTCCTCTGGAGCTGAAAG-3′ and 5′-GAACGTGTGGATCAGGTTGATGG-3′; ACTR2: 5′-GGTGTGACTCACATTTGCCCAG-3′ and 5′-TCAGCAGAGTGGTTGAAGGCGT-3′; MRTFA: 5′-CAGAACAGCACCTCACTGACTG-3′ and 5′-CAGGCAGTGATCGCAACTTCAG-3′

### 4.4. Western Blot

For Western blot analysis, total cellular protein lysates from each group were prepared using RIPA lysis buffer (25 mM Tris·HCl pH 7.6, 150 mM NaCl, 1% NP-40, 1% sodium deoxycholate, 0.1% SDS) (Thermo Fisher Scientific, Waltham, MA, USA, 89901), and protein concentrations were quantified by BCA assay. Protein samples were separated by SDS-PAGE gel electrophoresis and then transferred onto PVDF membranes (Cytiva, Marlborough, MA, USA, 10600021). Non-specific binding sites were blocked by incubating the membrane for 1 h at room temperature in blocking buffer containing 5% non-fat dried milk in 1× Tris-buffered saline (TBS). Subsequently, membranes were incubated individually with primary antibodies at 4 °C overnight. Membranes were then washed twice for 10 min each with 1× TBS containing 0.05% Tween-20 (TBS-T). Following washes, membranes were incubated with appropriate horseradish peroxidase-conjugated secondary antibodies diluted in blocking buffer for 1 h at room temperature. Membranes were again washed twice for 10 min each with TBS-T. Band signal intensity was captured using an enhanced chemiluminescence and colorimetric detection kit (AbClon, Seoul, Republic of Korea, ABC-3001) and quantified using Multi Gauge software (version 3.0, FUJIFILM).

Antibodies of GFP (Santa Cruz Biotechnology, Dallas, TX, USA, sc-9996), ARPC2 (Abcam, Cambridge, MA, USA, ab133315), COL4A1 (Cell Signaling Technology, Danvers, MA, USA, 50273), ACTA2 (Abcam, Cambridge, MA, USA, ab5694), MRTFA (Cell Signaling Technology, Danvers, MA, USA, 97109), ACTR2 (Abcam, Cambridge, MA, USA, ab47654), GAPDH (Bio-Rad, Hercules, CA, USA, MCA4740), goat anti-rabbit IgG (EMD Millipore, Burlington, MA, USA, AP132P), and goat anti-mouse IgG antibody (EMD Millipore, Burlington, MA, USA, AP124P) were used in the study.

### 4.5. Immunocytochemistry

MRC-5 cells grown on coverslips were fixed with 4% paraformaldehyde and permeabilized with 0.1% Triton X-100 for 10 min at room temperature. Coverslips were washed three times in TBS-T (0.1% Tween 20) and blocked in a drop of 1% BSA for 1 h prior to immunostaining. Primary antibody incubations with anti-MRTFA (Proteintech, Rosemont, IL, USA, 21166-1-AP) and GAPDH (Bio-Rad, Hercules, CA, USA, MCA4740) were performed overnight at 4 °C, followed by three washes in 0.1% TBS-T. Incubations with fluorochrome-labeled secondary antibodies were performed in Antibody diluent reagent solution (Invitrogen, Waltham, MA, USA, 003218) for 1 h at room temperature. Anti-ACTA2-FITC (Sigma-Aldrich, St. Louis, MO, USA, F3777) was incubated for 1 h at room temperature. Nuclei were counterstained with Hoechst (Thermo Fisher Scientific, Waltham, MA, USA, H3570) for visualization. Finally, cells were mounted using mounting media and visualized using a fluorescence microscope (Leica, Wetzlar, Germany, STELLARIS5).

### 4.6. In-Situ Proximity Ligation Assay (PLA)

In-situ PLA was performed using Naveni^®^ TriFlex Cell MR kit (Navinci, Uppsala, Sweden, TF.MR.100) according to the manufacturer’s instructions. Cells were incubated overnight at 4 °C with anti-MRTFA antibody (Proteintech, Rosemont, IL, USA, 21166-1-AP) and anti-Actin antibody (Sigma-Aldrich, St. Louis, MO, USA, A2228). Secondary antibody incubation, ligation, and amplification reactions, as well as Hoechst counterstaining, were performed as per the manufacturer’s instructions. Finally, cells were mounted using mounting media and visualized using a fluorescence microscope (Leica, Wetzlar, Germany, STELLARIS5).

### 4.7. Image Processing and Automated Quantification of Immunocytochemistry and PLA

Image analysis was performed using custom scripts written in Python version 3.9 utilizing the scikit-image, SciPy, and NumPy libraries. For all quantitative analyses, at least three independent fields of view were processed per specimen.

To quantify the nuclear accumulation of MRTFA, a global segmentation pipeline was developed to normalize target protein intensity against DNA content across the entire field of view., The Hoechst channel (nuclear marker) was smoothed using a Gaussian filter (sigma = 3.0) and global thresholding was applied using the Li method to adapt to varying image lighting, generating a binary mask of all nuclei within the image. Small objects (<700 pixels, likely noise) were excluded using the remove_small_objects function. The Nuclear Enrichment Index was calculated for each image as the ratio of the mean MRTFA fluorescence intensity to the mean Hoechst intensity within the total segmented nuclear area.

In-situ PLA signals were quantified by counting discrete puncta normalized to the nuclei count. Images were pre-processed by thresholding background noise (pixel intensity < 80) and applying a Gaussian blur (sigma = 1.5) followed by thresholding using Li’s method. PLA puncta were segmented with size exclusion (<25 pixels) to remove non-specific noise. Overlapping puncta were separated using watershed segmentation. Nuclei were segmented concurrently using parameters (Gaussian blur, sigma = 1.5; size exclusion, <3000 pixels), and the PLA index was calculated as the total number of PLA puncta divided by the total number of nuclei per field.

### 4.8. Statistical Analysis of Western Blot, qRT-PCR and Image Analysis

Quantified measurement of Western blot and qRT-PCR was statistically tested by One-way ANOVA using GraphPad Prism 8.0.

Statistical analyses of quantified image data were performed using the R version 4.3.1 powered by RStudio (2023.06.1+524) [45]. A generalized linear mixed model (GLMM) was fitted using the bglmer function with a Gamma distribution and square-root link function to model the quantification [46]. The model included treatment group as a fixed effect and both intra-specimen image variability and inter-experiment variability as random intercepts to account for the nested structure of the data. Estimated marginal means were computed using the emmeans package, and pairwise contrasts were performed to the control group. *p*-values were adjusted for multiple comparisons using the False Discovery Rate (FDR) method.

### 4.9. Bulk RNA-seq Data Processing and Analysis

RNA-seq reads were trimmed using TrimGalore (v0.6.10; parameters: --phred33 --fastqc_args --stringency 1 --length 20 --error 0.1), and ribosomal RNA sequences were removed using SortMeRNA (v4.3.6; parameters: --paired_out --out2 --fastx). The resulting non-rRNA reads were used as input for transcript quantification. Quantification was performed against the GRCh38.p13 (GENCODE) reference transcriptome using Salmon (v1.10.3; parameters: --libType A --validateMappings --numBootstraps 200 --dumpEq --dumpEqWeights --rangeFactorizationBins 4 --seqBias --writeUnmappedNames). Statistical analyses of the quantified data were subsequently performed using R (v4.5.0) powered by RStudio (2025-04-11). To improve the quality and speed of the analysis, genes with low expression (total counts < 2 × number of samples) or with an average transcript length ≤ 20 bp (mostly short non-coding RNAs) were removed prior to analysis. Genes located in the pseudoautosomal region of the Y chromosome (suffix “_PAR_Y”) were renamed by removing the suffix; if duplicate gene names resulted, their counts were summed.

### 4.10. Differential Expressed Gene Identification

Normalization was applied to correct for variations in sequencing depth and RNA composition across samples. Size factors were estimated iteratively to ensure convergence and robustness in the presence of heterogeneous expression distributions. Dispersion parameters were estimated using an empirical Bayes shrinkage approach. A local mean–variance fitting strategy was employed to accurately capture gene-specific variability, allowing flexible modeling of non-linear dispersion trends typically observed in complex transcriptomic datasets.

Differentially expressed genes were identified via likelihood ratio tests (LRT) comparing full and reduced models from DESeq2 (1.48.1), thereby evaluating whether inclusion of the experimental condition significantly improved model fit. Subsequently, Wald tests were performed to assess the significance of individual model coefficients, yielding log2 fold change estimates and confidence intervals for each contrast. Multiple testing correction was performed using both independent hypothesis weighting (IHW), which enhances detection power by incorporating expression-level covariates, and the Benjamini–Hochberg FDR procedure to control for type I error. Genes with a absolute log2 fold change over than 1 and an FDR less than 0.01 were considered significantly differentially expressed.

### 4.11. Pathway and Gene Set Analysis

Gene Ontology analysis was performed using the ‘enrichGO’ function from the clusterProfiler R package (parameters: pAdjustMethod = “BH”, pvalueCutoff = 0.01, qvalueCutoff = 0.05) on differentially expressed genes identified by DESeq2. When the number of differentially expressed genes exceeded 200, only the top 200 genes ranked by adjusted *p*-value were used for analysis.

### 4.12. Figure Preparation

Figures were assembled and labeled using Adobe Illustrator.

## Data Availability

The raw data supporting the conclusions of this article will be made available by the authors on request.

## Supplementary Materials

The following supporting information can be downloaded at: https://www.mdpi.com/article/10.3390/ijms27062729/s1.

## Abbreviations

The following abbreviations are used in this manuscript:

ACTA2	α-smooth muscle actin
ACTR2	actin-related protein 2
ARP2/3	actin-related protein 2/3
ARPC2	actin-related protein 2/3 complex subunit 2
ARPC4	actin-related protein 2/3 complex subunit 4
ATP10A	ATPase phospholipid transporting 10A
COL1A1	collagen type I alpha 1 chain
COL4A1	collagen type IV alpha 1 chain
CTGF	connective tissue growth factor
DEG	differentially expressed gene
EGFP	enhanced green fluorescent protein
EMT	epithelial-mesenchymal transition
FBS	fetal bovine serum
FBLN2	fibulin 2
FDR	false discovery rate
FMT	fibroblast-to-myofibroblast transition
FOXF1	forkhead box F1
G-actin	globular actin
GAPDH	glyceraldehyde-3-phosphate dehydrogenase
GLMM	generalized linear mixed model
GO	Gene Ontology
GSVA	Gene Set Variation Analysis
IHW	independent hypothesis weighting
IPF	idiopathic pulmonary fibrosis
IQR	interquartile range
LRRC32	leucine rich repeat containing 32
LRT	likelihood ratio test
MEM	minimum essential medium
MRC2	mannose receptor C type 2
MRTFA	myocardin-related transcription factor-A
NLS	nuclear localization signal
PDGF	platelet-derived growth factor
PLA	proximity ligation assay
qPCR	quantitative polymerase chain reaction
RNA-seq	RNA sequencing
SD	standard deviation
shRNA	small hairpin RNA
SRF	serum response factor
TAGLN	transgelin
TAZ	transcriptional coactivator with PDZ-binding motif
TGF-β	transforming growth factor-β
TNF-α	tumor necrosis factor-alpha
TPM1	tropomyosin 1
TRITC	tetramethylrhodamine
TSPAN2	tetraspanin 2
YAP	Yes-associated protein
