# Supplementary material for: ARPC2 Promotes Pulmonary Fibrosis by Regulating MRTFA Activity Independent of the Canonical ARP2/3 Complex
Source: Int J Mol Sci. 2026 Mar 17;27(6):2729. doi: 10.3390/ijms27062729 (PMC13027156; doi:10.3390/ijms27062729)
Supplement: Supplementary file 1 [file ijms-27-02729-s001.zip › ijms-4122887-supplementary/ijms-4122887-supplementary/ijms-4122887 supplimentary information.pdf]

Supplementary Information

ARPC2 Promotes Pulmonary Fibrosis by Regulating MRTFA Activity Independent of the Canonical ARP2/3 Complex

Eun Jo Du[1], Seo-Gyeong Bae[1], Hyunseong Kim[1], Sihyeon An[2*] and Kanghyun Ryoo[1*]

*These authors jointly supervised this work: Sihyeon An and Kanghyun Ryoo

Correspondence: Kanghyun Ryoo, E-mail: kh.ryoo@sapiensbio.com; Sihyeon An, E-mail: sh.an@ydgls.com

Running title: ARPC2 Modulates MRTFA to Induce Pulmonary Fibrosis

Supplementary Materials and Methods

Immunocytochemistry and quantification of ACTA2 stress fiber

Immunocytochemistry of ACTA2 was performed according to the protocol in Materials and Methods (main manuscript), followed by anti-ACTA2-FITC (Sigma-Aldrich, St. Louis, MO, USA, F3777) incubation for 1 hour at room temperature.

To quantify ACTA2 stress fiber formation level in image, ACTA2 intensity was collected in a whole image with normalization by GAPDH intensity. For each specimen, at least three images were quantified.

In-situ proximity ligation assay (PLA) of MRTFA with ARPC2, ARPC4 and ACTR2.

In-situ PLA was performed according to the protocol in Materials and Methods (main manuscript). To detect proximity of MRTFA and ARPC4, antibody against MRTFA (Santa Cruz Biotechnology, Dallas, TX, USA, sc-398675) and ARPC4 (Abcam, Cambridge, MA, USA, ab217065) were used.

In-vitro binding assay

Recombinant GST-MRTFA(N)-HA protein (2-269 a.a.) and anti-HA agarose bead (Thermo Fisher Scientific, Waltham, MA, USA, 26181) were dissolved in an in-vitro binding buffer [50 mM Tris-Cl (pH 8.0), 100 mM NaCl, 0.2 mM $CaCl_2$, 0.1 mM EDTA, 0.1 mM DTT and 1X protease/phosphatase inhibitor cocktail (GenDEPOT, Baker, TX, P3300)] and incubated for 30 mins at 4 ºC with gentle rocking. Recombinant His-ARPC2 was added and incubated for 1 hour at 4 ºC while gentle rocking. The beads were washed 3 times with wash buffer [in-vitro binding buffer added with 0.5% Triton X-100], then remaining proteins were eluted in 2X Lammeli's buffer [125 mM Tris-Cl (pH 6.8), 20% Glycerol, 2% SDS, 2% β-mercaptoethanol

and 0.02% Bromophenol blue].

Supplementary Figures Legends

Supplementary Figure 1. Attenuation of stress fiber formation by ARPC2. (a) Left panel shows representative immunofluorescence images of ACTA2 (green) and GAPDH (red) in ARPC2 or ACTR2 knockdown cells. Quantified ACTA2 intensity (right panel) was normalized to GAPDH and compared to TGF-β1-treated group. Box plots show median, IQR, and 1.5× IQR whiskers; individual data points are jittered (n=4; $^{*}P_{adj} < 0.05$; $^{**}P_{adj} < 0.01$). (b) Representative immunofluorescence images of ACTA2 (green) and GAPDH (red) in MRC-5 cells. ACTA2 was detected using Alexa Fluor 555-conjugated antibody, which is shown in pseudo color green. Quantified ACTA2 intensity is normalized to GAPDH intensity. Box plots show median, interquartile range (IQR), and 1.5× IQR whiskers; individual data points are jittered (n=6; $^{**}P_{adj} < 0.01$; compared to control).

Supplementary Figure 2. ARPC2 and MRTFA interaction dynamics upon TGF-β1 stimulation. (a) Schematic diagram of MRTFA domains, highlighting the NLS region (marked as green) between second and third region of the three RPEL motifs (marked as orange). (b) *In situ* PLA to measure interaction between MRTFA and ARPC2, ARPC4 or ACTR2, respectively, in MRC-5 cells after 72-hour TGF-β1 stimulation under serum-starved conditions. PLA puncta (red) counts normalized to the number of nuclei (Hoechst; blue). Box plots show median, IQR, and 1.5× IQR whiskers; individual data points are jittered (n=4; $^{**}P_{adj} < 0.01$; compared to control). (c) *In vitro* binding assay using recombinant His-ARPC2 and GST-MRTFA(N)-HA (2-269 a.a.) to demonstrate direct interaction between ARPC2 and RPEL motifs region of MRTFA.

Supplementary Figure 3. Differential gene expression between ARPC2, ACTR2 and MRTFA knockdown transcriptome upon TGF-β1 stimulation. Differential gene expression

(DEG) between each (a) ARPC2, (b) ACTR2 and (c) MRTFA knockdown cells vs. TGF-β1-treated control cells and (d) ARPC2 vs. ACTR2 were analyzed and displayed volcano plot (left panel) and Gene Ontology (GO) enrichment analysis of every top 200 DEGs (right panel).
