# Supplementary figures and images for: ARPC2 Promotes Pulmonary Fibrosis by Regulating MRTFA Activity Independent of the Canonical ARP2/3 Complex
Source: Int J Mol Sci. 2026 Mar 17;27(6):2729. doi: 10.3390/ijms27062729 (PMC13027156; doi:10.3390/ijms27062729)

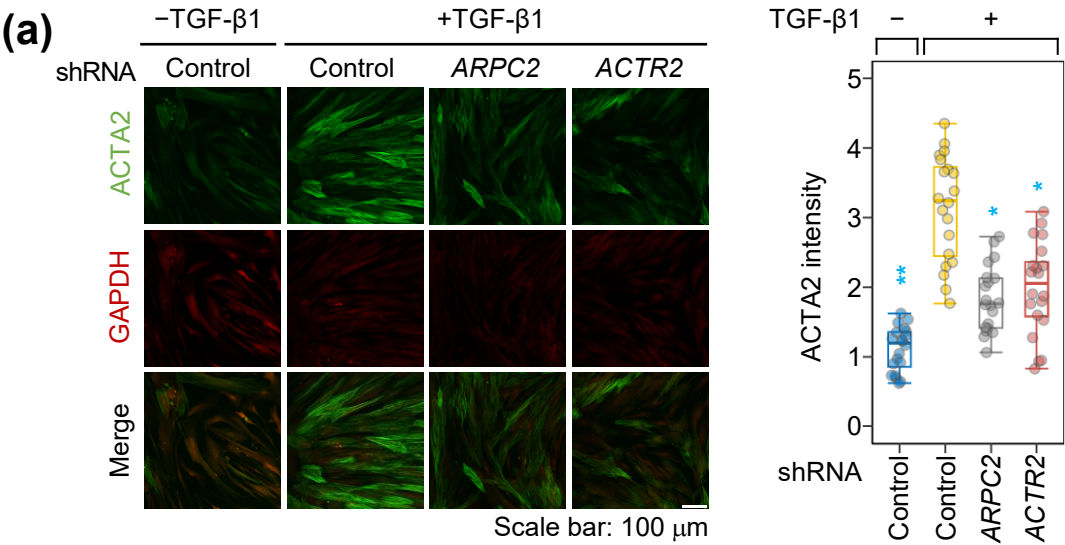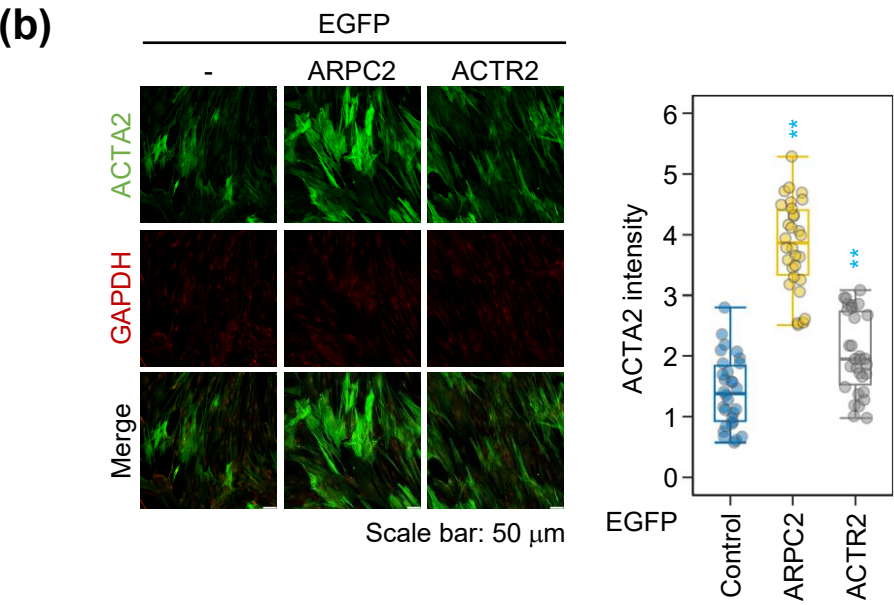

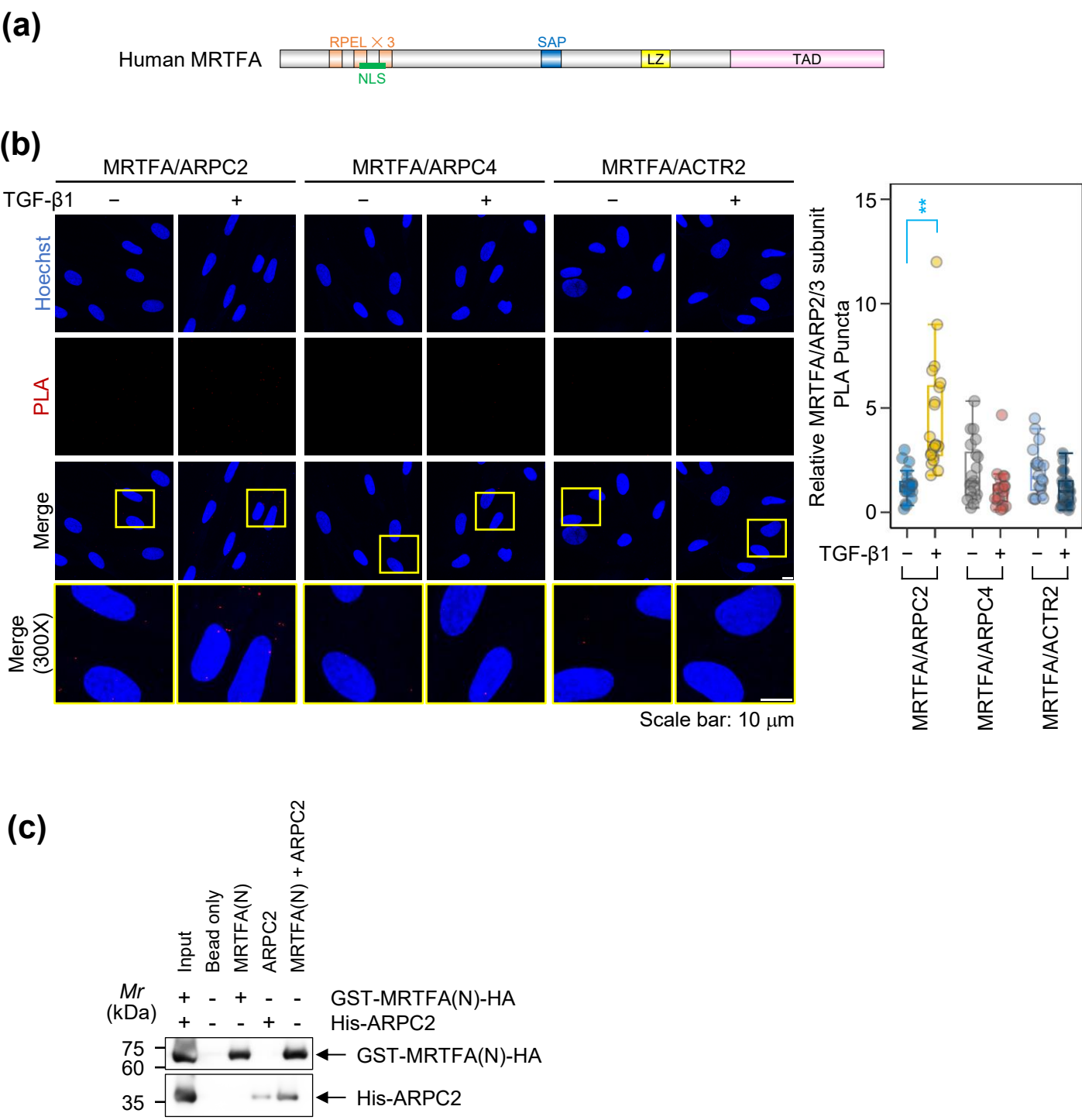

Supplementary Figure 3

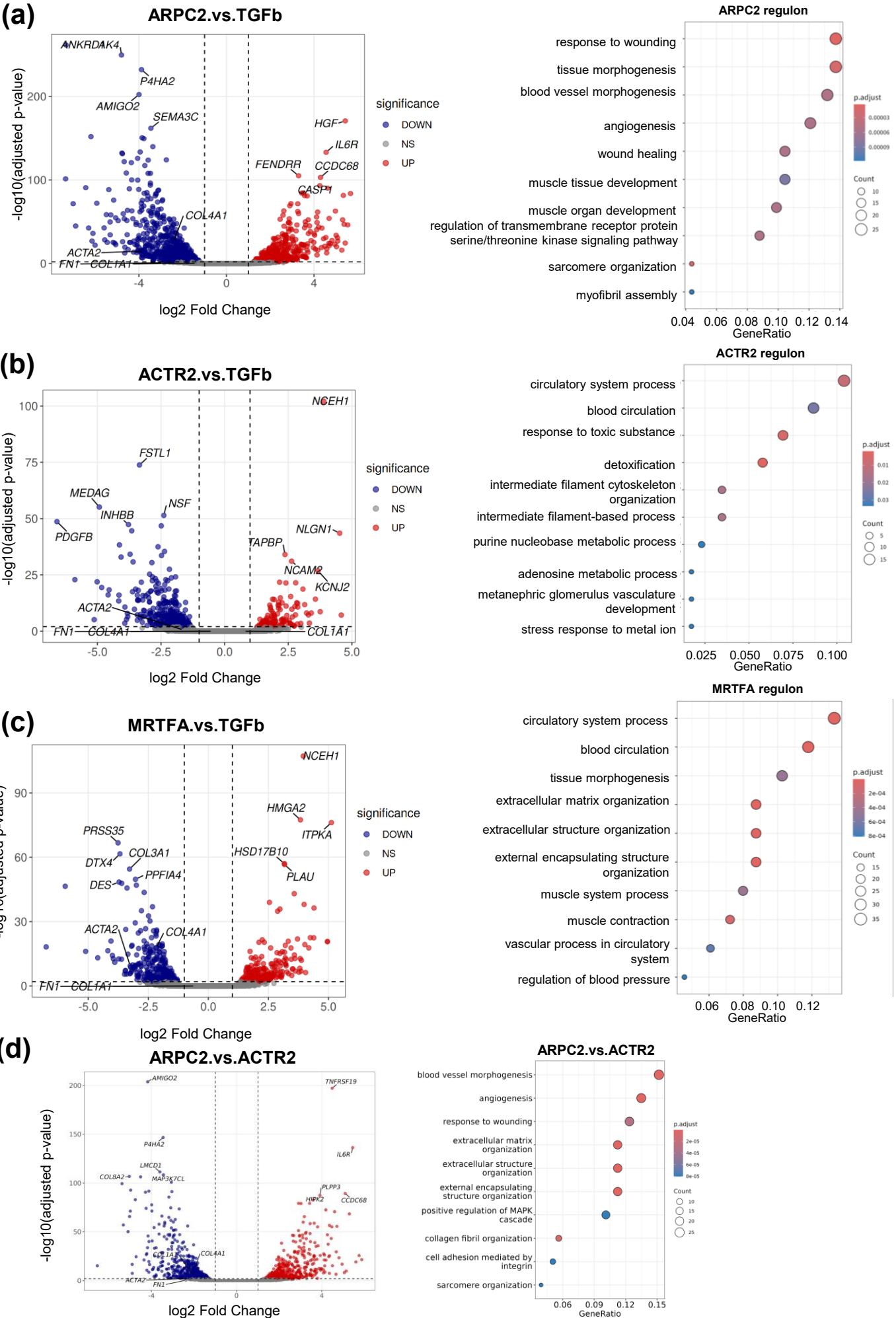

Supplement: Supplementary file 1 [file ijms-27-02729-s001.zip › ijms-4122887-supplementary/ijms-4122887-supplementary/ijms-4122887-rev1 supplementary figure.pdf]
